# Efficacy of technology-based personalised feedback on diet quality in young Australian adults: results for the advice, ideas and motivation for my eating (Aim4Me) randomised controlled trial

**DOI:** 10.1017/S1368980023000253

**Published:** 2023-06

**Authors:** Rebecca L Haslam, Jennifer N Baldwin, Kristine Pezdirc, Helen Truby, John Attia, Melinda J Hutchesson, Tracy Burrows, Robin Callister, Leanne Hides, Billie Bonevski, Deborah A Kerr, Sharon I Kirkpatrick, Megan E Rollo, Tracy A McCaffrey, Clare E Collins

**Affiliations:** 1 School of Health Sciences, College of Health, Medicine and Wellbeing, University of Newcastle, University Drive, Callaghan 2308, Australia; 2 Priority Research Centre for Physical Activity and Nutrition, University of Newcastle, Callaghan, Australia; 3 Hunter Medical Research Institute, New Lambton Heights, Australia; 4 School of Human Movement and Nutrition Sciences, University of Queensland, Australia; 5 School of Medicine and Public Health, College of Health, Medicine and Wellbeing, University of Newcastle, Callaghan, Australia; 6 School of Biomedical Sciences and Pharmacy, College of Health, Medicine and Wellbeing, University of Newcastle, Callaghan, Australia; 7 School of Psychology, University of Queensland, Brisbane, Australia; 8 School of Population Health, Faculty of Health Sciences, Curtin University, Perth, Australia; 9 Curtin Health Innovation Research Institute, Curtin University, Perth, Australia; 10 School of Public Health Sciences, University of Waterloo, Waterloo, ON, Canada; 11 Department of Nutrition, Dietetics and Food, Monash University, Notting Hill, Australia

**Keywords:** Young adults, Diet, Nutrition therapy, Randomised controlled trial, eHealth

## Abstract

**Objective:**

Web-based dietary interventions could support healthy eating. The Advice, Ideas and Motivation for My Eating (Aim4Me) trial investigated the impact of three levels of personalised web-based dietary feedback on diet quality in young adults. Secondary aims were to investigate participant retention, engagement and satisfaction.

**Design:**

Randomised controlled trial.

**Setting:**

Web-based intervention for young adults living in Australia.

**Participants:**

18–24-year-olds recruited across Australia were randomised to Group 1 (control: brief diet quality feedback), Group 2 (comprehensive feedback on nutritional adequacy + website nutrition resources) or Group 3 (30-min dietitian consultation + Group 2 elements). Australian Recommended Food Score (ARFS) was the primary outcome. The ARFS subscales and percentage energy from nutrient-rich foods (secondary outcomes) were analysed at 3, 6 and 12 months using generalised linear mixed models. Engagement was measured with usage statistics and satisfaction with a process evaluation questionnaire.

**Results:**

Participants (*n* 1005, 85 % female, mean age 21·7 ± 2·0 years) were randomised to Group 1 (*n* 343), Group 2 (*n* 325) and Group 3 (*n* 337). Overall, 32 (3 %), 88 (9 %) and 141 (14 %) participants were retained at 3, 6 and 12 months, respectively. Only fifty-two participants (15 % of Group 3) completed the dietitian consultation. No significant group-by-time interactions were observed (*P* > 0·05). The proportion of participants who visited the thirteen website pages ranged from 0·6 % to 75 %. Half (Group 2 = 53 %, Group 3 = 52 %) of participants who completed the process evaluation (Group 2, *n* 111; Group 3, *n* 90) were satisfied with the programme.

**Conclusion:**

Recruiting and retaining young adults in web-based dietary interventions are challenging. Future research should consider ways to optimise these interventions, including co-design methods.

Young adults have lower diet quality compared with all other age groups^(1)^. In Australia, only 2·5 % of males and 5·7 % of females aged 18–24 years meet the recommended daily intake of vegetables (5–6 servings/d)^(2)^. For young adults, poor diet increases their risk for CVD, type 2 diabetes and certain cancers^(3)^. Barriers to healthy eating include time constraints, limited food preparation skills, peer influences and lower cost and greater accessibility of energy-dense nutrient-poor foods^(4)^.

Dietary interventions targeting dietary improvement in young adults are needed. A systematic review of twenty-four nutrition intervention studies among university/college students found no high-quality studies^(5)^. Twelve of these studies reported improvements in dietary intake, including three with an improvement in diet quality, and four of twelve studies targeting weight achieved weight loss^(5)^. Another systematic review of fifty-four randomised controlled trials (RCT) investigating behaviour change techniques for improving dietary intake of young adults reported a median sample size of 162 participants (range 37–2343), with 74 % of studies having ≤3 months follow-up^(6)^. A review of eight poor to moderate quality trials of weight management in young women found that five studies reported significant differences in weight change over time periods ranging from 10 weeks to 1 year, with dietary outcomes (energy density) reported in only one study^(7)^.

Adopting and maintaining positive dietary behaviour change requires individualised dietary feedback, monitoring and support^(8)^. A systematic review of forty-five intervention trials identified that brief, tailored dietary interventions are effective in improving dietary behaviours;^(9)^ however, further research is required to determine whether improvements can be sustained in the long term. Goal setting and personalised feedback are the most frequently used behaviour change techniques for improving dietary intake among young adults^(6)^. Goal setting involves the client and clinician agreeing on a goal that is defined in terms of the behaviour in question (e.g. *increase serves of vegetables by one serve/d)*, while feedback refers to the monitoring of goals and behaviours and provision of informative or evaluative feedback *(*e.g. frequency, or quantity of intake of vegetables*)* ^(10)^.

Dietary interventions also need to consider the optimal mode of delivery, with many young adults not wanting to talk about the weight on social media^(11)^. Web-based technologies are evolving to support health for young adults, offering specialised platforms for written, audio and video information that is accessible using mobile devices. Web-based interventions could therefore be an effective, time-efficient mode to engage young adults. One study has shown that a computerised weight loss intervention helped individuals set behaviour change goals, and that the integration of counselling resulted in greater weight loss^(8)^. However, the impact of different levels of feedback and goal-setting interventions delivered via web-based technologies on dietary patterns of young adults has not been investigated. The aim of the current study was to investigate the impact of three levels of personalised dietary feedback, nutrition education and goal setting, using web-based technologies, on diet quality (Australian Recommended Food Score (ARFS), primary outcome) in young adults over 12 months. Secondary aims were to investigate participant retention, engagement and satisfaction.

## Methods

### Trial design

The Advice, Ideas and Motivation for My Eating (Aim4Me) was a 12-month prospective, randomised, open, blinded endpoint trial. Potential participants were recruited from across Australia, then screened using a web-based eligibility survey. Those interested and eligible provided electronic informed consent and contact details. Login details and passwords were then sent, allowing access to the study website and baseline assessment questionnaires. After completion of the baseline demographic survey, participants were randomised into one of three dietary intervention groups: brief dietary feedback (Group 1); comprehensive dietary feedback and website (Group 2); comprehensive feedback and website plus dietetic consultation (Group 3). Participants were prompted to complete the baseline Australian Eating Survey (AES) after randomisation, with those who did not complete the AES excluded from the analysis. Group 1 (control) received a *brief* personalised feedback report on their diet quality only, based on the Healthy Eating Quiz (HEQ). Group 2 received a *comprehensive* feedback report based on their Australian Eating Survey (AES) FFQ data compared to food and nutrient recommendations^(12)^, plus access to the *Aim4Me* website, which included resources to support healthy eating, goal setting and self-monitoring. Group 3 had access to a 30-min video consultation with an Accredited Practising Dietitian in addition to the Group 2 elements. Ethics approval for the *Aim4Me*trial was granted by the University of Newcastle Human Research Ethics Committee (H-2017–0087). The *Aim4Me*trial was prospectively registered with the Australian New Zealand Clinical Trials Registry (ACTRN#12618000325202). Reporting was consistent with the Consolidated Standards of Reporting Trials (CONSORT) guidelines (see online Supplemental file 1) and the Template for Intervention Description and Replication (TIDieR) checklist (see online Supplemental file 2)^(13,14)^.

### Participants

Eligibility criteria included: aged between 18 and 24 years, had a BMI ≥ 18·5 kg/m^2^ (calculated in the eligibility screen using self-reported height and weight), lived in Australia, were not pregnant or planning pregnancy in the next year, had no medical conditions and no diagnosis of current or previous eating disorder, were not studying/previously studied a nutrition degree and had access to a smartphone and computer/tablet, access to the Internet and an active email account.

Participants were recruited nationally across Australia using social media platforms (paid targeted ads on Facebook and Instagram) and from universities, organisations and communities who interact with young adults via links and flyers, as well as local and national media releases through newspapers, magazines and radio stations. Potential participants were invited to visit the *Aim4Me*home page, which contained information about the study and a link to the eligibility survey where participants could check their eligibility and sign up for the study if eligible. Email invitations were distributed to contacts who had previously signed up for notifications on nutrition-related studies. Snowballing, whereby participants could share a link to the *Aim4Me*home page with friends and colleagues inviting them to take part, was also used. A sample size of 2570 young adults was targeted^(15)^. Incentives were based on a review of other research studies using incentives, adjusting based on the views of young adults and consistency with ethical guidance for payment of participants in research. An incentive, including donations to OzHarvest (https://www.ozharvest.org/), a food rescue organisation that provides meals to people in need, was offered to encourage participants to complete follow-up surveys. To further increase survey completion, a $50 grocery voucher was offered for completion of each of the 6-month and 12-month surveys.

### Randomisation

Eligible participants were randomly allocated (1:1:1) to one of the three groups. Randomisation occurred in random permuted blocks of varying size and was stratified by postcode location (using the Monash Modified Model)^(16)^, sex and BMI (18·5–24·9 *v*. ≥ 25 kg/m^2^). Randomisation was coded by an independent statistician who provided the coding to the software developers to programme the web-based environment. The research team did not have access to the randomisation code.

### Interventions

#### Group 1 (control): brief diet quality feedback

Group 1 participants were provided with a link to complete the HEQ (http://healthyeatingquiz.com.au/) at baseline, 3, 6 and 12 months. The HEQ is a short (5-min) web-based diet quality assessment tool that provides *brief* feedback on diet variety based on the core nutrient-rich food groups^(17)^. The HEQ is modelled on the ARFS, with scoring algorithms providing total ARFS and subscale scores (fruit, vegetables, grains, dairy, meat, meat alternatives, water, extras)^(18,19)^. Group 1 participants were provided with a brief personalised report generated from the HEQ to identify key areas for improving their diet quality (e.g. increase variety of vegetables consumed regularly). These participants also completed the Australian Eating Survey (AES) as part of their study assessments but did not receive the more detailed AES personalised feedback report.

#### Group 2: comprehensive feedback on nutritional adequacy + Aim4Mewebsite nutrition resources

Participants in Group 2 were given access to the *Aim4Me*website for 12 months. Each participant had access to their own unique *Aim4Me*dashboard which was accessible using their participant log-in details provided after randomisation. The dashboard contained links to the four main components of the website: comprehensive personalised dietary assessment and feedback (using the AES); healthy eating resource materials; goal setting and self-monitoring. More detailed descriptions of these components are provided in Supplemental file 3. Dietary feedback was provided following completion of the AES, which is an automated, web-based, 120-item FFQ that assesses usual food and nutrient intakes in adults^(20)^. Participants were provided with a real-time *comprehensive* personalised report that compared usual dietary intake to national food and nutrient recommendations (% energy from core, nutrient-rich food groups and energy-dense, nutrient-poor food groups) and age and sex-specific Nutrient Reference Values^(21)^ (% energy from protein, fat, saturated fat, carbohydrate; daily grams of fibre, seven minerals and five vitamins). The AES report provides a total diet quality score and diet quality sub-scale scores (see below ‘Outcome measures’). Participants were encouraged to set goals for improving their diet quality (increasing nutrient-rich core foods, decreasing energy-dense nutrient-poor foods) based on their AES report. Participants could set goals by selecting from a pre-set list of goals derived from consensus based on talking to young adults (e.g. *increase fruit intake to 2 serves/d*) or by developing their own. The pre-set list of goals was designed as SMART (Specific-Measurable-Achievable-Realistic-Timely) goals in keeping with behaviour change principles (see online Supplemental File 3). Participants could select up to three goals to focus on at any one time. Participants in Group 2 received the report but no further support on the interpretation of the report for setting dietary goals. For self-monitoring, participants were prompted by email and text to self-monitor their goals by going to their dashboard. Participants were asked to reflect on how well they had achieved their goals, and how important each goal was to them (5-point Likert scale). Based on these responses, participants were either provided with generic feedback or further information to support them in achieving their goals (see online Supplemental File 3).

#### Group 3: dietitian consultation + group 2 elements

Participants randomised to Group 3 also had access to the *Aim4Me*website and were also offered a single 30-min video consultation with an Accredited Practising Dietitian. Upon receiving their personalised AES feedback report at baseline, participants were prompted via an automated email to book their dietitian consultation within 14 d. Participants selected a consultation time that suited them best and were assigned to a dietitian that was available for that preferred consultation time. In the structured consultation sessions, the dietitian reviewed the goals set by the participant relative to the AES report and helped develop strategies to address self-identified barriers and facilitators to healthy eating. To personalise the sessions prior to the appointment, participants were asked to complete a brief self-administered Personalised Nutrition Questionnaire^(22)^ informed by the Behaviour Change Wheel theory^(23)^. In completing the Personalised Nutrition Questionnaire, participants were asked to self-identify and prioritise eighteen factors (capability = 7, opportunity = 5 and motivation = 6) that they perceived to affect their ability to achieve healthy eating. Dietitians used the Personalised Nutrition Toolbox^(22)^, which contains intervention strategies mapped to each factor of the Personalised Nutrition Questionnaire and the behaviour change techniques required to personalise strategies to address the participant’s individual goals. Dietitians were trained in the consultation protocol to ensure consistency in delivery, including how to use the online platform, interpreting the AES and reviewing dietary goals and strategies. Seven dietitians delivered the consultation over the study period.

### Assessments

Socio-demographic characteristics were collected at baseline via an online survey administered through the *Aim4Me*website. Age, gender, country of birth, Aboriginal/Torres Strait Islander status, highest qualification, annual income, marital status and living arrangements were collected. Level of socio-economic advantage/disadvantage was assessed using participants’ residential postcode to calculate the Socio-Economic Index for Areas Index of Relative Socioeconomic Disadvantage percentiles (SEIFA IRSD)^(24)^. Food insecurity was evaluated using a single question asking if in the past 12 months they had run out of food and could not afford to buy food (Yes/No). Frequency and duration of physical activity were assessed using the seven-item Godin Leisure-Time Exercise tool, from which the amount of moderate-vigorous physical activity in min/week was calculated^(25)^. Frequency of alcohol intake was categorised as ‘Never’, ‘Monthly or less’, ‘2–4 times a month’, ‘2–3’ times a week’ or ‘4+ times a week’^(26)^. Current smoking status was categorised as ‘Non-smoker’, ‘Less than once a week’, ‘At least once a week’ or ‘Daily’, with the latter three categories collapsed as ‘Current smoker’^(27)^. BMI (kg/m^2^) was calculated using participants’ self-reported weight and height^(28)^ and categorised according to WHO recommendations: <18·5 kg/m^2^, 18·5–24·99 kg/m^2^, 25–29·99 kg/m^2^ or ≥30 kg/m^2(29)^.

### Outcome measures

The primary outcome was diet quality at baseline and 3, 6 and 12 months, assessed using the ARFS. Secondary outcomes included ARFS subscales (vegetables, fruit, meat, meat alternatives, grains and dairy foods) and percentage energy (%E) from nutrient-rich core foods. Participants were sent up to three email reminders over a 10-d period to complete the AES at each time point. Participants were invited to complete the AES by clicking on the link provided in the reminder email or by following the link on their unique trial dashboard. The ARFS is based on seventy questions within the AES using the core food groups recommended in the Australian Dietary Guidelines^(12)^. An ARFS total score (0–73 points) and eight subscales were calculated: vegetables (twenty questions), fruit (twelve questions), meat (seven questions), meat alternatives (six questions), grains (twelve questions), dairy foods (ten questions), water (one question) and extras (two questions e.g. spreads and sauces). Participants who were vegetarian were assigned zero points for meat questions and double points for vegetarian options consumed ‘at least once/week’, plus an additional point if both ‘soybeans, tofu’ and ‘other beans, lentils’ were consumed ‘at least once/week’. Higher ARFS values indicate better diet quality^(30)^. The ARFS has previously demonstrated adequate validity against an FFQ and plasma and skin carotenoids as biomarkers of fruit and vegetable intake in adults^(18,30,31)^. From the AES, the percentage contribution to total energy intake (%E) contributed by specific food groups was calculated for nutrient-rich core foods as the secondary outcome (e.g. fruits, vegetables, grains).

Outcomes related to the secondary aims included engagement, retention and satisfaction. Engagement was measured using study website usage statistics. Engagement measures included completion of the HEQ (Group 1 only), the number of logins to the website, clicks on resources and links, views of personalised dietary feedback and views and completion of goal setting and tracking (Groups 2 and 3). Engagement was also measured by use of the dietitian consultation (Group 3). Retention was assessed as the proportion of participants who completed the AES at 3, 6 and 12 months. Satisfaction with study components was evaluated at 3, 6 and 12 months using a process evaluation questionnaire developed by the research team. Questions were a combination of Likert scales and open questions. Participants were sent email reminders to complete the process evaluation questionnaire on the *Aim4Me* website, as well as a direct link to complete the survey through Qualtrics (Provo). Group 1 was asked about their satisfaction with the brief feedback provided by the HEQ. For Groups 2 and 3, questions covered the comprehensive, personalised dietary feedback, resources on the website, goal setting and tracking and overall satisfaction. Group 3 was also asked about their satisfaction with the dietitian consultation.

### Statistical methods

Data were analysed using IBM SPSS Statistics 27 (IBM Corp.). Demographic and baseline characteristics were reported for participants across the three study groups as mean (sd) for continuous variables and percentages (counts) for categorical variables.

Generalised linear mixed models were used to analyse the primary outcome (ARFS total) and secondary outcomes (ARFS subscales, percentage energy from nutrient-rich core foods) for the impact of the treatment group (Group 1 control *v*. Group 2 *v*. Group 3), time (baseline, 3, 6 and 12 months) and the treatment-by-time interaction. Group, time and the group × time interaction formed the three terms for the base model, ensuring that outcomes for participants who were lost to follow-up at 3, 6 or 12 months were retained in the mixed model analyses, consistent with an ‘intention-to-treat’ approach. Base models were initially tested using compound symmetry and unstructured variance types, and the appropriate variance type (i.e. yielding the lowest Akaike’s Information Criterion) was selected for each model. Coefficients and *P*-values for the treatment-by-time interaction term were examined to determine the efficacy of the intervention using a significance level of *P* < 0·05 for all outcomes.

A per-protocol sensitivity analysis was also conducted to examine the impact of the AES comprehensive feedback report and *Aim4Me* website intervention components only on diet quality. Participants in Group 3 who did not take part in the dietitian video consultation were identified and combined with participants in Group 2, as these participants received only the comprehensive feedback report and *Aim4Me* website access. Generalised linear mixed models were then used to analyse the primary outcome (ARFS) and secondary outcomes (ARFS subscales and percentage energy nutrient-rich core foods) for the impact of treatment of Groups 2 and 3 combined (comprehensive feedback report and *Aim4Me* website only) *v*. Group 1 control (brief feedback report).

For analysis of process evaluation data, categorical variables were reported as percentages (counts).

As the intervention did not change beyond the initial 3 months and given the low numbers of respondents at each timepoint, process evaluation data were pooled such that one response for each participant was included, with the earliest response included for those participants who responded at more than one timepoint.

## Results

### Recruitment

**Fig. 1:**
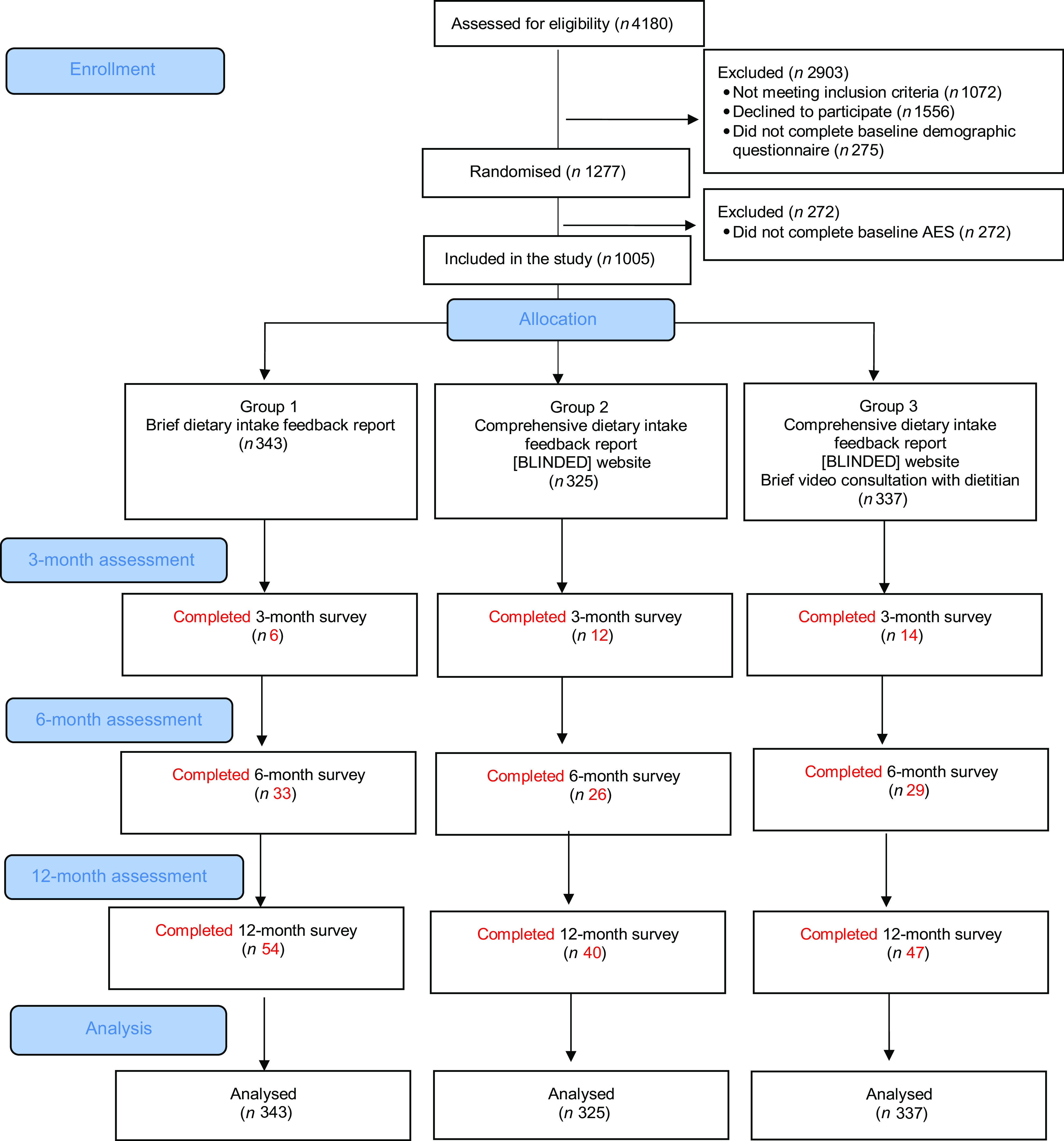
CONSORT diagram describing study design and flow of participants through the 12-month *Aim4Me* randomised controlled trial

Figure 1 shows the CONSORT diagram describing study design and flow of participants through the 12-month *Aim4Me* RCT. Of the 4180 participants who expressed interest by starting the baseline demographic survey on the trial dashboard, 1277 were eligible and randomised into the study, of whom 1005 completed the baseline *Aim4Me* surveys and were included in the study (Group 1 *n* 343; Group 2 *n* 325, Group 3 *n* 337) (Fig. 1). Recruitment commenced in March 2018 and ceased December 2019. Recruitment ceased before the targeted sample size of 2570 participants was reached due to funding deadlines. Data collection ceased in December 2020, 12 months after the final participant was recruited.

### Participant characteristics

Participant characteristics are provided in Table 1. Of the 1005 participants randomised into the study, the majority (85 %) were female, and the mean age was 21·7 ± 2·0 years. Mean diet quality score (total ARFS) was 33·6 ± 10·2 points out of a possible 73 points, and %E from nutrient-rich core foods was 66·4 ± 13·7 %. Additional information regarding the representativeness of the sample is provided in Supplemental file 4.

**Table 1:** Characteristics of participants enrolled at baseline in the *Aim4Me* trial (*n* 1005)

	Group 1 Control: brief report on diet quality *n* 343		Group 2 Website + comprehensive dietary feedback *n* 325		Group 3 Video consult + website + comprehensive dietary feedback *n* 337	
	Mean	sd	Mean	sd	Mean	sd
Age, years	21·8	1·9	21·6	1·9	21·8	1·9
	*n*	%	*n*	%	*n*	%
Gender, female	84	289	85	275	86	291
Country of birth, Australia	83	286	85	275	83	281
Aboriginal and/or Torres Strait Islander	2	5	2	8	2	7
	Mean	sd	Mean	sd	Mean	sd
SEIFA index of relative socioeconomic disadvantage*	62·5	26·1	63·7	26·1	61·4	26·9
	*n*	%	*n*	%	*n*	%
Highest qualification						
University/Higher university degree	38	130	32	104	34	113
Certificate/Diploma/Trade/Apprenticeship	13	46	10	32	13	45
Higher school certificate/School certificate or lower	47	160	57	186	51	173
School certificate or lower	2	7	1	3	2	6
Employment status						
Full time paid employment	23	78	16	53	21	71
Part time paid employment	20	69	24	77	23	76
Casual employment	30	104	43	138	36	122
Self-employed/Other paid employment	4	14	2	8	3	9
Not currently in paid employment	23	78	15	49	18	59
Annual income (AUD)						
No income	9	29	5	17	8	28
< $20 800	34	115	43	140	36	120
$20 800–$31 199	19	66	19	60	22	73
$31 200–$51 999	18	63	14	45	15	51
≥$52 000	13	343	12	38	13	44
Don’t know/Don’t want to answer	7	24	8	25	6	21
Food insecurity in past 12 months†	11	37	10	33	14	48
Marital status						
Married/ de facto	14	48	13	43	18	61
Never married	86	294	87	281	81	273
Separated/divorced	0·3	1	0·3	1	1	3
	Mean	sd	Mean	sd	Mean	sd
MVPA (min/week)	233	235	251	228	247	249
	*n*	%	*n*	%	*n*	%
Smoking status						
Never	90	307	93	301	88	297
< Once/week	7	23	5	15	7	25
> Once/week	2	6	1	2	2	7
Daily	2	7	2	7	2	8
Smoked ≥100 cigarettes in lifetime	12	42	10	31	10	35
	Mean	sd	Mean	sd	Mean	sd
Weight (kg)	70·1	16·1	70·3	14·7	70·7	14·9
BMI (kg/m^2^)	24·7	4·8	24·5	4·5	24·8	4·8
% energy nutrient-rich core foods	66·3	13·2	66·2	14·0	66·7	14·0
% energy EDNP foods	33·7	13·2	33·8	14·0	33·3	14·0
ARFS‡	32·6	10·3	33·5	10·0	31·8	10·2
ARFS vegetable	12·5	5·1	12·8	4·9	12·1	5·1
ARFS fruit	5·0	3·0	5·4	3·1	4·8	3·0
ARFS grains	5·4	2·1	5·7	2·2	5·4	2·2
ARFS dairy	3·3	1·8	3·4	1·8	3·4	1·9
ARFS meat	2·3	1·6	2·2	1·6	2·2	1·5
ARFS meat alternatives	2·5	1·5	2·5	1·5	2·4	1·4

ARFS, Australian recommended food score; AUD, Australian dollars; EDNP foods, energy-dense nutrient-poor foods; MVPA, moderate to vigorous physical activity; SEIFA, socio-economic indexes for areas.

* Higher percentile corresponds with greater socio-economic advantage.

† Classified as a time in the past 12 months when one ran out of food and could not afford to buy more.

‡ Scored from 0 to73 points, higher score indicates better diet quality.

### Participant retention

Participant retention was low, with 32 (3 %) participants across the sample completing the 3-month follow-up AES, 88 (9 %) completing the 6-month follow-up AES and 141 (14 %) completing the 12-month follow-up AES. Retention at 3, 6 and 12 months was 2 %, 10 % and 16 % for Group 1, 4 %, 8 % and 12 % for Group 2, and 4 %, 9 % and 14 % for Group 3, respectively. There were no baseline differences in socio-demographic characteristics, diet quality or BMI between those participants who were lost to follow-up and those who were retained in the study. In Group 3, fifty-two (15 %) eligible participants completed the dietitian consultation. Comparing the fifty-two participants who completed the dietitian consultation with the remainder of Group 3 (*n* 285), there were no differences in age, gender, socio-economic advantage, food security, BMI, baseline ARFS, smoking status, physical activity or alcohol consumption (*P* > 0·05).

### Diet quality outcomes

Overall, no significant (*P* > 0·05) group-by-time interactions were observed for ARFS (primary outcome), ARFS subscales or percentage of energy from core foods (secondary outcomes) (Table 2). At 3 months, participants in Group 3 had higher percentage energy from core foods (mean (95 % CI) difference between groups 12·8 (2·0, 23·6), *P* = 0·021); however, this difference appears to be due in large part to a decline in Group 1 rather than a substantial improvement in diet quality in Group 3; there were no consistent or sustained differences at the 6- or 12-month follow-up.

**Table 2:** Mean (95 % CI) change in primary (ARFS) and secondary outcomes within groups and between groups (intention-to-treat population) over time in the Aim4Me trial

Outcome	Month	Change from baseline, mean						Difference between groups						*P*-value
		Group 1§ control: brief report *n* 343		Group 2‖ website + comprehensive feedback *n* 325		Group 3¶ video consult + website + comprehensive feedback *n* 337		Group 2 *v*. group 1		Group 3 *v*. group 1		Group 2 *v*. group 3		
		Mean	95 % CI†	Mean	95 % CI†	Mean	95 % CI†	Mean	95 % CI‡	Mean	95 % CI‡	Mean	95 % CI‡	
ARFS	3	−4·15	–10·31, 2·01	2·43	–2·03, 6·88	−0·28	–4·36, 3·80	6·58	–1·04, 14·19	3·87	–3·53, 11·27	−2·71	–8·76, 3·35	0·374
	6	0·41	–2·25, 3·06	2·52	–0·48, 5·51	0·15	–2·68, 2·98	2·11	–1·90, 6·12	−0·25	–4·14, 3·64	−2·63	–6·50, 1·77	
	12	1·47	–0·67, 3·60	−0·20	–2·66, 2·26	−0·33	–2·60, 1·94	−1·66	–4·93, 1·60	−1·80	–4·92, 1·33	−0·13	–3·49, 3·23	
ARFS Vegetable	3	−0·65	–3·66, 2·35	1·08	–1·09, 3·25	0·64	–1·35, 2·63	1·73	–1·98, 5·45	1·29	–2·32, 4·90	−0·44	–3·39, 2·51	0·830
	6	1·12	–0·18, 2·41	0·98	–0·48, 2·44	0·14	–1·24, 1·52	−0·13	–2·09, 1·82	−0·98	–2·88, 0·92	−0·85	–2·86, 1·17	
	12	0·93	–0·12, 1·97	0·24	–0·96, 1·44	0·34	–0·76, 1·45	−0·69	–2·28, 0·91	−0·58	–2·11, 0·94	0·10	–1·53, 1·74	
ARFS Fruit	3	−1·70	–3·60, 0·20	0·06	–1·31, 1·44	0·04	–1·22, 1·30	1·76	–0·59, 4·11	1·74	–0·55, 4·03	−0·02	–1·89, 1·85	0·621
	6	−0·37	–1·19, 0·46	0·11	–0·81, 1·04	−0·46	–1·34, 0·42	0·48	–0·76, 1·72	−0·09	–1·30, 1·11	−0·57	–1·85, 0·70	
	12	−0·03	–0·69, 0·63	−0·41	–1·17, 0·35	−0·24	–0·94, 0·46	−0·38	–1·39, 0·63	−0·21	–1·17, 0·76	0·17	–0·87, 1·21	
ARFS Meat	3	−0·27	–1·29, 0·76	0·37	–0·37, 1·11	−0·20	–0·87, 0·48	0·64	–0·63, 1·09	0·07	–1·16, 1·29	−0·57	–1·57, 0·43	0·182
	6	0·03	–0·41, 0·47	0·01	–0·49, 0·51	−0·17	–0·65, 0·30	−0·02	–0·68, 0·65	−0·20	–0·85, 0·44	−0·19	–0·87, 0·50	
	12	0·11	–0·25, 0·46	0·00	–0·41, 0·41	−0·58	–0·95, –0·20	−0·11	–0·65, 0·44	−0·68	–1·20, –0·16*	−0·58	–1·13, –0·02*	
ARFS Meat alternatives	3	−0·20	–1·13, 0·72	0·01	–0·66, 0·68	−0·38	–1·00, 0·23	0·21	–0·93, 1·36	−0·18	–1·29, 0·93	−0·39	–1·30, 0·52	0·337
	6	0·26	–0·15, 0·66	0·61	0·16, 1·06	−0·04	–0·46, 0·39	0·35	–0·25, 0·96	−0·29	–0·88, 0·30	−0·64	–1·27, –0·02*	
	12	0·25	–0·08, 0·57	−0·02	–0·39, 0·35	0·20	–0·14, 0·54	−0·26	–0·75, 0·23	−0·05	–0·52, 0·42	0·22	–0·29, 0·72	
ARFS Grains	3	−0·97	–2·50, 0·56	0·38	–0·73, 1·48	−0·53	–1·54, 0·49	1·35	–0·54, 3·23	0·44	–1·39, 2·28	−0·90	–2·40, 0·60	0·531
	6	0·36	–0·31, 1·02	1·10	0·35, 1·84	0·61	–0·10, 1·31	0·74	–0·26, 1·74	0·25	–0·72, 1·22	−0·49	–1·52, 0·54	
	12	0·05	–0·48, 0·58	0·14	–0·47, 0·75	0·38	–0·19, 0·94	0·09	–0·72, 0·90	0·33	–0·45, 1·11	0·24	–0·60, 1·07	
ARFS Dairy	3	−0·19	–1·45, 1·07	0·30	–0·61, 1·20	0·23	–0·61, 1·06	0·48	–1·07, 2·04	0·41	–1·10, 1·92	−0·07	–1·30, 1·16	0·125
	6	−0·66	–1·20, –0·11	−0·18	–0·80, 0·43	0·37	–0·21, 0·95	0·48	–0·35, 1·30	1·03	0·23, 1·83*	0·56	–0·29, 1·40	
	12	0·26	–0·18, 0·70	−0·02	–0·52, 0·49	−0·13	–0·59, 0·34	−0·28	–0·94, 0·39	−0·39	–1·03, 0·25	−0·11	–0·80, 0·58	
% energy nutrient-rich core foods	3	−6·65	–15·63, 2·34	2·21	–4·27, 8·69	6·12	0·17, 12·07	8·85	–2·24, 19·95	12·76	1·97, 23·56*	3·91	–4·90, 12·72	0·416
	6	2·21	–1·67, 6·10	0·97	–3·40, 5·35	0·94	–3·20, 5·08	−1·24	–7·10, 4·62	−1·28	–6·96, 4·41	−0·04	–6·07, 6·00	
	12	0·66	–2·46, 3·77	0·53	–3·07, 4·12	−0·42	–3·74, 2·89	−0·13	–4·89, 4·64	−1·08	–5·64, 3·48	−0·95	–5·85, 3·95	

ARFS, Australian recommended food score.

* *P* < 0·05.

† Time differences were calculated as (3 months minus baseline), (6 months minus baseline) and (12 months minus baseline).

‡ Between-group differences in changes from baseline to 12 months.

§ Number of participants in Group 1 who completed 3, 6 and 12-month follow-up surveys: *n* 6, *n* 33, *n* 48, respectively.

‖ Number of participants in Group 2 who completed 3, 6 and 12-month follow-up surveys: *n* 12, *n* 26, *n* 40, respectively.

¶ Number of participants in Group 3 who completed 3, 6 and 12-month follow-up surveys: *n* 14, *n* 29, *n* 47, respectively.

### Sensitivity analyses

Results for the per-protocol sensitivity analyses comparing participants in Group 1 (*n* 343) *v*. Groups 2 and 3 combined (*n* 610) are shown in Table 3. There were no significant group-by-time interactions for ARFS, ARFS subscales or percentage of energy from core foods (*P* > 0·05). Participants in the combined Groups 2 and 3 had a higher ARFS at 3 months (mean (95 % CI) between-group difference (7·2 (0·1, 14·3) points, *P =* 0·048) and percentage energy from core foods (11·3 % (0·9, 21·8), *P =* 0·033) compared to those in Group 1. However, this again appears to be driven largely by a decline in Group 1 rather than a substantial improvement in Groups 2 and 3; there was no difference between groups at the 6- or 12-month follow-up (*P* > 0·05).

**Table 3:** Per-protocol sensitivity analysis investigating mean (95 % CI) change in primary (ARFS) and secondary outcomes within groups and between groups comparing Group 1 (control- brief feedback report) with Groups 2 and 3 combined (website + comprehensive feedback report only)

Outcome	Month	Change from baseline				Difference between groups		*P*-value
		Group 1 control: brief report *n* 343		Groups 2 and 3 combined website + comprehensive feedback only§ *n* 610		Groups 2 and 3 combined *v*. group 1		
		Mean	95 % CI†	Mean	95 % CI†	Mean	95 % CI‡	
ARFS	3	−5·11	–11·38, 1·16	2·07	–1·28, 5·41	7·17	0·06, 14·29*	0·126
	6	−0·56	–3·36, 2·24	1·85	–0·32, 4·01	2·41	–1·14, 5·95	
	12	0·50	–1·81, 2·80	0·43	–1·43, 2·28	−0·07	–3·03, 2·89	
ARFS Vegetable	3	−1·05	–3·99, 1·89	1·24	–0·33, 2·81	2·29	–1·04, 5·63	0·607
	6	0·72	–0·60, 2·03	0·87	–0·14, 1·89	0·15	–1·51, 1·82	
	12	0·53	–0·55, 1·61	0·69	–0·18, 1·56	0·16	–1·23, 1·55	
ARFS Fruit	3	−1·66	–3·58, 0·26	0·31	–0·72, 1·33	1·96	–0·21, 4·14	0·313
	6	−0·33	–1·19, 0·54	0·03	–0·64, 0·69	0·35	–0·74, 1·44	
	12	0·01	–0·70, 0·72	−0·02	–0·59, 0·55	−0·03	–0·94, 0·89	
ARFS Meat	3	−0·36	–1·41, 0·69	0·13	–0·43, 0·69	0·49	–0·71, 1·68	0·517
	6	−0·07	–0·54, 0·41	−0·07	–0·44, 0·30	−0·002	–0·60, 0·60	
	12	0·01	–0·38, 0·40	−0·28	–0·59, 0·04	−0·29	–0·79, 0·21	
ARFS Meat alternatives	3	−0·30	–1·26, 0·66	−0·18	–0·70, 0·33	0·11	–0·98, 1·21	0·945
	6	0·16	–0·27, 0·59	0·32	–0·02, 0·65	0·16	–0·39, 0·71	
	12	0·15	–0·21, 0·51	0·16	–0·13, 0·45	0·01	–0·45, 0·47	
ARFS Grains	3	−1·27	–2·82, 0·29	0·004	–0·83, 0·84	1·27	–0·49, 3·03	0·246
	6	0·06	–0·66, 0·77	0·77	0·22, 1·32	0·71	–0·19, 1·62	
	12	−0·25	–0·84, 0·35	0·14	–0·34, 0·61	0·39	–0·38, 1·15	
ARFS Dairy	3	−0·56	–1·85, 0·72	0·44	–0·25, 1·13	1·00	–0·46, 2·46	0·010*
	6	−1·03	–1·62, –0·45	0·10	–.36, 0·55	1·13	0·39, 1·87*	
	12	−0·12	–0·60, 0·37	−0·12	–0·51, 0·27	0·05	–0·63, 0·62	
% energy nutrient-rich core foods	3	−6·37	–15·55, 2·81	4·97	0·07, 9·87	11·34	0·92, 21·8*	0·193
	6	2·50	–1·66, 6·66	1·98	–1·23, 5·19	−0·52	–5·78, 4·75	
	12	0·94	–2·50, 4·38	1·05	–1·71, 3·81	0·11	–4·31, 4·52	

ARFS, Australian recommended food score.

* *P* < 0·05.

† Time differences were calculated as (3 months minus baseline) and (6 months minus baseline) and (12 months minus baseline).

‡ Between-group differences in changes from baseline to 12 months.

§ Excludes participants in Group 3 who completed the dietitian video consultation.

### Participant engagement

Engagement with the *Aim4Me* website (Groups 2 and 3) was poor. Across the thirteen main pages of the website, the proportion of participants who visited pages (unique page views) ranged from 0·6 % (Articles) to 75 % (Set goals). The top five most frequently visited pages were: (1) Set goals (75 %); (2) Track goals (47 %); (3) Theme of the Month (34 %); (4) View goals (34 %) and (5) AES feedback report (28 %). The average time spent on these five pages was approximately 50 s per page. The top five goal-setting pages visited were: (1) Vegetables and salad (7 %); (2) Fruit (4 %); (3) Confectionary (2·5 %); (4) Fried and takeaway (2 %) and (5) Baked sweet products (2 %). The top five visited Themes of the Month were ‘Munch your way through March’ which covered tips for increasing vegetable and fruit intake (*n* 61 visits), ‘New Year, New You’ which covered goal setting and changing habits (*n* 49), ‘Shape up for Summer’ which covered how to avoid fad diets and setting healthy challenges (*n* 27), ‘How to fuel an active lifestyle’ which covered topics such as carbohydrate, sports drinks, protein, muscle cramps and supplements (*n* 24), and finally ‘Mind your budget’ which covered how to eat on a budget, meal planning and cost-saving tips for Christmas (*n* 24).

### Participant satisfaction

Feedback was provided by Group 1 (*n* 102), Group 2 (*n* 111) and Group 3 (*n* 90 for the website; *n* 33 for the dietitian consultation) (see online Supplemental file 5). The following percentages relate to those completing the process evaluation, not to the whole cohort. In Group 1, most participants (73 %) were satisfied with the brief feedback provided by the Healthy Eating Quiz. Approximately half (53 % and 52 % in Groups 2 and 3, respectively) of participants were satisfied with the *Aim4Me* program overall. Satisfaction rates were highest for the AES report (70 % and 84 %). Approximately half of the participants were satisfied with the ‘Theme of the month’ (46 % and 54 % for Groups 2 and 3, respectively), ‘My goals’ (40 % and 74 %) and ‘Tracking goals’ (46 % and 60 %) features.

In Group 3, process evaluation data were available for thirty-three participants (63 %) who completed the dietitian consultation. Most (82 %) reported that the video platform was easy to use and that the picture and sound quality were acceptable (85 % and 79 %, respectively). Most felt that the dietitian was relatable (82 %), easy to understand (88 %) and professional (82 %). Participants mostly reported that the advice was motivating (70 %), relevant (76 %), personalised (76 %) and helped them make changes to their eating habits (67 %). Reasons for not attending the consultation included not knowing about the consultation (*n* 4), feeling that the advice from the Australian Eating Survey report was sufficient (*n* 6), not being able to make the appointment (*n* 2) or unable to access the video consultation system (*n* 2).

## Discussion

Intention-to-treat analyses found no significant group-by-time interaction for diet quality outcomes in this RCT of three levels of personalised dietary feedback for young adults. Recruiting and retaining young adults over the 12-month intervention was a challenge. Web-based interventions tailored to young adults require further investigation as a strategy to engage them in improving dietary patterns.

While our study attracted over 4000 and recruited over 1000 young adults, we did not reach the sample size required. Reporting of recruitment and retention in young adult nutrition and physical activity interventions is generally poor, with less than half of 107 RCT adequately reporting recruitment^(32)^. In our study, we could have used a targeted and paid social media campaign from the outset of the study to improve recruitment, as it was implemented approximately 7 months after recruitment began. Future studies could also consider social networking platforms to increase engagement with potential participants, as young adults prefer social media as a means for researchers to provide one-way communication^(11)^.

The characteristics of the young adults enrolled in the *Aim4Me* study were different from the characteristics of young adults reported previously, except for diet quality. Participants in the current study were more active than in previous reports^(33)^. However, physical activity levels in the current study were self-reported unlike levels reported by Howie et al which were measured using accelerometers. Overall diet quality was poor, which is consistent with previous reports which have identified that young adults have poor diet quality compared to other adult age groups^(1)^. Intake of energy-dense, nutrient-poor foods also highlighted the sub-optimal diet quality of these participants. Intake of energy-dense, nutrient-poor foods made up approximately a third of total energy intakes; however, this is consistent with data from the most recent national nutrition survey of the Australian population^(34)^. Additionally, males were under-represented, with 85 % of participants being female. Females tend to be more health conscious and more interested in eating healthfully^(35)^, which may explain why a greater number of females enrolled. A survey of Finnish young adults reported that females (18–35 years) are significantly more likely to engage and be involved in seeking health-related information compared to males^(36)^. Additionally, previous studies have reported a lower recruitment of males in dietary interventions in comparison to females^(35,37)^. Development of health promotion programmes should take into account the current gender differences in recruitment. For example, considering the use of gender-targeted recruitment strategies and materials in future interventions in order to engage more males.

In this study, only 3 %, 9 % and 14 % of participants were retained at 3, 6 and 12 months, respectively. Additionally, less than one-third of participants in Groups 2 and 3 accessed the personalised dietary feedback from the AES. In a systematic review of fifty-four RCT investigating behaviour change techniques for improving dietary intake of young adults, the mean retention rate was 78 % (range 22–98 %)^(6)^. While the majority of included studies were short term, with 74 % having ≤3 months follow-up, and the median sample size was 162 participants (range 37–2343), there are likely further reasons for our comparatively poor retention. We did not pre-specify any stopping rules for the trial based on participant attrition; this could be considered for future trials where retention is anticipated to be a challenge. Our surveys took approximately 45 min to complete and were not embedded in the *Aim4Me* website, which likely affected participation. We initially offered donations to OzHarvest (a food rescue organisation that donates meals to people in need) as an incentive to complete surveys; however, a more effective strategy was the offer of a $50 gift card reimbursement, which saw higher survey completion at 6 and 12 months. Previous systematic reviews have shown that financial incentives are the most successful for retaining young adults^(32)^ and that personalised electronic feedback support greater retention in e-health interventions targeting young adults^(38)^. Therefore, to successfully engage and retain young adults, the reward needs to outweigh the effort of participation. Future studies could also consider tailoring health promotion strategies to the differing psycho-behavioural characteristics of young adults to increase engagement and uptake^(39)^.

Technology-based interventions have rapidly entered the health research setting but few studies report the unintended consequences of study implementation. In our study, many participants did not engage with the website, complete surveys or attend dietitian consultations. While we are unable to ascertain whether these problems were user-related (e.g. participants lacked motivation) or a design issue (e.g. the website was difficult to navigate), they were exacerbated by technical issues. The *Aim4Me* website platform was developed and managed by external web developers. This introduced difficulties in communication, timeliness and quality. Reminders were sent to complete surveys via email. However, IT failures meant that the external scheduling platform often failed to send these reminders. Several participants commented in the process evaluation that they would have liked more reminders to engage with the website. In the future, these could be sent across additional modalities (e.g. SMS) to address this issue. Our website analytics also did not have the capability to track usage data by intervention group. Other issues have been identified, such as software updates, poor user interface and network issues^(40)^. There is a need for methodological protocols to ensure consistent and effective use of technology in health research. We also suggest involving young adults in co-designing the content and selecting the platform to ensure that they are relevant and appropriate.

Another unexpected finding was the low uptake of dietitian consultations (15 % of Group 3 participants). Young adults more frequently source their nutrition information from online resources (93 %) compared to healthcare professionals (5 %)^(41)^, despite the majority of young adults (87 %) believing that health professionals provide reliable information. In our study, the personalised feedback in the AES report might have been sufficient, as suggested by six participants in the process evaluation. This is supported by the diet quality scores in our study being similar to a previous study reporting diet quality scores of 27 977 Australians aged 16–24 years (33·6 ± 10·2 points compared with 34·5 ± 9·3 points, respectively)^(17)^. We also relied on participants to book the dietitian consultation; in future, the dietitian could initiate the consultation. Additionally, participants were not able to choose their dietitian, which may have affected their decision to book an appointment, as they were unable to choose a dietitian of their choice. Our findings are consistent with previously observed barriers to accessing dietitian support, including fear of judgement, low confidence, mistrust and relying on word of mouth to make decisions regarding health services^(42)^.

Participants who did use the dietitian consultation generally provided positive feedback. Due to the conflicting nutrition information found online and considering dietitians’ skills in facilitating dietary behaviour change, it is important for nutrition professionals to bridge this gap with young adults and assist them in accessing professional advice. A systematic review has found that counselling by Accredited Practising Dietitians confers annual savings of $830 to $1893 per person, with fewer medications and hospital admissions related to chronic diet-related conditions^(43)^, although these savings could be more relevant to older adults.

Adding tailored feedback and counselling to digital interventions can increase their effectiveness in improving eating behaviours, although further support is likely required to assist young adults to achieve long-term dietary improvements^(44)^. We found no significant changes in dietary intake or differences between groups over 12 months, although we did not reach the sample size needed to detect a difference. There is a variety of factors that influence one’s ability to adhere to dietary change, including environmental, socio-cultural and psychological influences^(45)^. Young adults may be more interested in the ‘here and now’ rather than their longer term health, and it is possible that healthy eating was not a priority due to balancing the demands of studying and working and perceived lack of money, time and knowledge^(4)^. For an individual to succeed with long-term dietary change, they likely need ongoing support for the substantial cognitive resources required to improve dietary behaviours and address structural barriers such as food affordability. Future research could use a co-design methodology to explore how peer–peer networking and relatable role models could be used to encourage young adults to support each other in healthy eating and learn strategies to improve diet quality. Recent reviews of co-design practices in nutrition research and in m-health studies have found that co-design techniques are used regularly and may be a useful strategy to consider in m-health setting, although none were specifically conducted with young adults and the effectiveness of the co-design methods required further evaluation^(46,47)^. Additionally, a review of social media for nutrition education and behaviour change found that eleven out of sixteen studies that included a social media component had a significant improvement in at least one nutrition outcome^(48)^. However, topics focusing on weight have been shown to have poor engagement with young adults and should be avoided or not used as the sole target^(11)^. The inclusion of co-design methodologies and use of social media is therefore likely to be useful strategies to consider in future studies to improve engagement of young adults in digital health trials^(49)^.

### Limitations

The high loss to follow-up needs to be considered when interpreting results. Process evaluation findings also need to be interpreted with caution as it is possible that satisfaction rates changed over time; however, this was not captured due to the high attrition rates. Dietary data were collected by self-report; therefore, misestimation of intake may have occurred. Males were underrepresented (15 %), meaning that generalisability to males or other age groups is limited. Compared with the national young adult population^(50)^, a greater proportion of participants were born in Australia and were in the healthy BMI range and a lower proportion of participants were daily smokers, further limiting generalisability (see online Supplemental file 4). Finally, the website analytics did not have the capability to track usage data by intervention group, which is a limitation and should ideally be included in future studies.

## Conclusions

In this randomised trial investigating three levels of web-based personalised dietary feedback, no differences were seen for dietary outcomes over 12 months. We experienced challenges with the recruitment, engagement and retention of young adults. Future research could investigate co-design methods to explore suitable content and delivery of technology-based dietary interventions, as well as approaches to optimise recruitment and retention for young adults.

## Supplementary material

For supplementary material accompanying this paper visit https://doi.org/10.1017/S1368980023000253. click here to view supplementary material
