# Supplementary material for: Efficacy of technology-based personalised feedback on diet quality in young Australian adults: results for the advice, ideas and motivation for my eating (Aim4Me) randomised controlled trial
Source: Public Health Nutr. 2023 Feb 9;26(6):1293–305. doi: 10.1017/S1368980023000253 (PMC10346011; doi:10.1017/S1368980023000253)
Supplement: Supplementary file 1 [file S1368980023000253sup001.zip › S1368980023000253sup001.docx]

**Supplementary file 3.** Frequency of responses to process evaluation questions for the Advice, Ideas and Motivation for My Eating (Aim4Me) study (*n*=1005). Data are shown as n (%).

| **Program element** | **Question** | **Group 1**  *Brief feedback report* | **Group 2**  *Comprehensive feedback report* | **Group 3**  *Video consult + comprehensive feedback* |
| --- | --- | --- | --- | --- |
| **Healthy Eating Quiz**  Group 1 n=102 | Overall, how would you rate your satisfaction with the Healthy Eating Quiz? |  |  |  |
|  | Somewhat satisfied/Very satisfied | 74 (73) | - | - |
|  | Neutral | 25 (24) | - | - |
|  | Somewhat dissatisfied/Very dissatisfied | 2 (2) | - | - |
|  | Have not completed | 1 (1) | - | - |
| **Aim4Me program^#^** | Overall, how would you rate your satisfaction with the Aim4Me program? |  |  |  |
|  | Somewhat satisfied/Very satisfied | - | 58 (53) | 45 (52) |
|  | Neutral | - | 45 (41) | 38 (44) |
|  | Somewhat dissatisfied/Very dissatisfied | - | 8 (7) | 3 (3) |
|  | Please rate your level of satisfaction with each of the following website features: |  |  |  |
|  | Theme of the month | - |  |  |
|  | Somewhat satisfied/Very satisfied | - | 21 (46) | 19 (54) |
|  | Neutral | - | 11 (24) | 7 (20) |
|  | Somewhat dissatisfied/Very dissatisfied | - | 2 (4) | 0 |
|  | Have not used | - | 12 (26) | 9 (26) |
|  | Australian Eating Survey personalized feedback report |  |  |  |
|  | Somewhat satisfied/Very satisfied | - | 39 (70) | 42 (84) |
|  | Neutral | - | 9 (16) | 4 (8) |
|  | Somewhat dissatisfied/Very dissatisfied | - | 2 (3) | 1 (2) |
|  | Have not used | - | 6 (11) | 3 (6) |
|  | My goals |  |  |  |
|  | Somewhat satisfied/Very satisfied | - | 19 (40) | 26 (74) |
|  | Neutral | - | 16 (33) | 5 (14) |
|  | Somewhat dissatisfied/Very dissatisfied | - | 4 (8) | 1 (3) |
|  | Have not used | - | 9 (19) | 3 (9) |
|  | Tracking goals |  |  |  |
|  | Somewhat satisfied/Very satisfied | - | 22 (46) | 21 (60) |
|  | Neutral | - | 9 (19) | 7 (20) |
|  | Somewhat dissatisfied/Very dissatisfied | - | 7 (15) | 2 (6) |
|  | Have not used | - | 10 (21) | 5 (14) |
| **Dietitian video consultation^#^** | The video platform was easy to use |  |  |  |
|  | Agree/ Strongly agree | - | - | 27 (82) |
|  | Neither agree nor disagree | - | - | 4 (12) |
|  | Disagree/ Strongly disagree | - | - | 2 (6) |
|  | The picture quality was acceptable |  |  |  |
|  | Agree/ Strongly agree | - | - | 28 (85) |
|  | Neither agree nor disagree | - | - | 5 (15) |
|  | Disagree/ Strongly disagree | - | - | 0 |
|  | The sound quality was acceptable |  |  |  |
|  | Agree/ Strongly agree | - | - | 26 (79) |
|  | Neither agree nor disagree | - | - | 5 (15) |
|  | Disagree/ Strongly disagree | - | - | 2 (6) |
|  | The dietitian was relatable |  |  |  |
|  | Agree/ Strongly agree | - | - | 27 (82) |
|  | Neither agree nor disagree | - | - | 5 (15) |
|  | Disagree/ Strongly disagree | - | - | 1 (3) |
|  | The dietitian was easy to understand |  |  |  |
|  | Agree/ Strongly agree | - | - | 29 (88) |
|  | Neither agree nor disagree | - | - | 4 (12) |
|  | Disagree/ Strongly disagree | - | - | 0 |
|  | The dietitian was professional |  |  |  |
|  | Agree/ Strongly agree | - | - | 27 (82) |
|  | Neither agree nor disagree | - | - | 6 (18) |
|  | Disagree/ Strongly disagree | - | - | 0 |
|  | I was able to build rapport with the dietitian |  |  |  |
|  | Agree/ Strongly agree | - | - | 26 (79) |
|  | Neither agree nor disagree | - | - | 7 (21) |
|  | Disagree/ Strongly disagree | - | - | 0 |
|  | The advice was motivating |  |  |  |
|  | Agree/ Strongly agree | - | - | 23 (70) |
|  | Neither agree nor disagree | - | - | 8 (24) |
|  | Disagree/ Strongly disagree | - | - | 2 (6) |
|  | The advice was relevant to me as a young adult |  |  |  |
|  | Agree/ Strongly agree | - | - | 25 (76) |
|  | Neither agree nor disagree | - | - | 8 (24) |
|  | Disagree/ Strongly disagree | - | - | 0 |
|  | The advice was personalised to me |  |  |  |
|  | Agree/ Strongly agree | - | - | 25 (76) |
|  | Neither agree nor disagree | - | - | 7 (21) |
|  | Disagree/ Strongly disagree | - | - | 1 (3) |
|  | The advice helped me set goals for Aim4Me |  |  |  |
|  | Agree/ Strongly agree | - | - | 24 (73) |
|  | Neither agree nor disagree | - | - | 8 (24) |
|  | Disagree/ Strongly disagree | - | - | 0 |
|  | The advice helped me to make changes to my eating habits |  |  |  |
|  | Agree/ Strongly agree | - | - | 22 (67) |
|  | Neither agree nor disagree | - | - | 8 (24) |
|  | Disagree/ Strongly disagree | - | - | 3 (9) |

^#^ Percentages have been calculated based on the number of valid responses for each question.
