# Supplementary material for: Efficacy of technology-based personalised feedback on diet quality in young Australian adults: results for the advice, ideas and motivation for my eating (Aim4Me) randomised controlled trial
Source: Public Health Nutr. 2023 Feb 9;26(6):1293–305. doi: 10.1017/S1368980023000253 (PMC10346011; doi:10.1017/S1368980023000253)
Supplement: Supplementary file 1 [file S1368980023000253sup001.zip › S1368980023000253sup004.docx]

**Supplementary file 2. Additional information regarding the Aim4Me sample**

**Recruitment:** Participants were recruited across Australia using social media platforms (Instagram, paid targeted ads on Facebook) and from universities, organisations and communities who interact with young adults via links and flyers, as well as local and national media releases through newspapers, magazines and radio stations. Email invitations were distributed to contacts who had previously signed up for notifications on nutrition-related studies. Snowballing, whereby participants could share a link to the trial dashboard with friends and colleagues inviting them to take part, was also utilised. An incentive was offered to encourage participants to complete follow-up surveys which included donations to OzHarvest, a food rescue organisation that provides meals to people in need, if participants completed the surveys. To increase survey completion toward the end of the study, a $50 grocery voucher was offered for completion of each of the 6-month and 12-month surveys.

**Representativeness:**

- 84% of participants were born in Australia (n=842), compared with 75% of Australians aged 15-24 years.^1^
- 2% of participants (n=20) identified as Indigenous, compared with 5% of the national population aged 15-24 years.
- 12% (n=118) had experienced food insecurity in the past 12 months, compared with an estimated 4-13% of the general Australian population.^2^
- 65% of participants were in the healthy weight range, compared with 50% of Australians aged 18-24 years.^3^
- 2% (n=22) of participants were daily smokers, compared with 9% of Australians aged 18-24 years who smoke daily.^4^
- 27% of participants (n=276) exceeded single occasion risk guidelines (4+ standard drinks on one occasion) at least monthly, compared with 41% of Australian adults aged 18-24 years.^4^
- Mean self-reported MVPA of participants was 243 ± 237 minutes per week, compared with median (25^th^, 75^th^) 27 minutes (16, 41) MVPA per day for women and 34 minutes (20, 52) MVPA per day for men (estimated 189 minutes and 238 minutes per week, respectively) in a sample of 475 Australians aged 22 years, collected by accelerometry.^5^
- Mean diet quality score (total ARFS) was 33.6 ± 10.2 points out of a possible 73 points, compared with 34.5 ± 9.0 points in a sample of 247 Australian adults aged 18+ years^6^ and 32.6 ± 8.8 points in a sample of 10,629 Australian women aged 50-55 years^7^
- Percentage of total energy from core foods was 66.4 ± 13.7%.

**How the analysed sample differed from the recruited sample:** Only participants who completed both the baseline demographic survey and the Australian Eating Survey (AES) were included in the study. As randomisation occurred after participants completed the demographic survey but before the AES, those participants who did not subsequently complete the AES were excluded from the study (n=272).

**How any missing data were handled:** We conducted generalised linear mixed models to analyse the data. This approach addresses missing data within the analysis and retains all participants in the analysis.

**References**

1. Australian Institute of Health and Welfare. Demographics of Australian young people and their families. <https://www.aihw.gov.au/reports/children-youth/australias-youth/contents/demographics#living-arrangements>. Published 2021. Accessed December 7, 2021.

2. Bowden M. *Understanding food insecurity in Australia.* 2020.

3. Australian Institute of Health and Welfare. Australia's youth: Body mass index. <https://www.aihw.gov.au/reports/children-youth/body-mass-index>. Published 2021. Accessed December 7, 2021.

4. Australian Institute of Health and Welfare. Alcohol, tobacco and other drugs in Australia. <https://www.aihw.gov.au/reports/phe/221/alcohol-tobacco-other-drugs-australia/contents/population-groups-of-interest/young-people>. Published 2021. Accessed December 7, 2021.

5. Howie EK, McVeigh JA, Winkler EAH, et al. Correlates of physical activity and sedentary time in young adults: the Western Australian Pregnancy Cohort (Raine) Study. *BMC Public Health.* 2018;18(1):916.

6. Pullen R, Kent K, Sharman MJ, Schumacher TL, Brown LJ. A Comparison of Diet Quality in a Sample of Rural and Urban Australian Adults. *Nutrients.* 2021;13(11):4130.

7. Baldwin JN, Forder P, Haslam R, et al. Change in diet quality over 12 years in the 1946-51 cohort of the Australian Longitudinal Study on Women's Health. *Nutrients.* 2020;12(1):147.
