# Supplementary material for: Efficacy of technology-based personalised feedback on diet quality in young Australian adults: results for the advice, ideas and motivation for my eating (Aim4Me) randomised controlled trial
Source: Public Health Nutr. 2023 Feb 9;26(6):1293–305. doi: 10.1017/S1368980023000253 (PMC10346011; doi:10.1017/S1368980023000253)
Supplement: Supplementary file 1 [file S1368980023000253sup001.zip › S1368980023000253sup005.docx]

**Supplementary File 1.** Description of the components of the Aim4Me website.

| **Personalized dietary feedback** | - Automated (computer generated) personalized feedback report on dietary intake will be available to access through the website, using the Australian Eating Survey. - The feedback report will be provided at baseline, 3, 6 and 12 months if the appropriate dietary assessment tool is completed. This will allow the individual to compare their previous reports and self-assess change. |
| --- | --- |
| **Healthy eating resource materials** | - A web-based resource library of evidence-based materials will include links to apps, articles, fact sheets, recipes and information related to healthy eating and targeting motivators and barriers to behavior change expressed by young males and females, e.g., in relation to the key barriers of cost and time, the website will include resource tips on eating healthy on a budget, quick and easy meals, and budget recipes. - Other accessible content will include ‘Theme of the month’ which provides educational information on a new topic each month (e.g. Love your Heart is May content), ‘Food’ which provides short snippets of information on specific food groups e.g. how to eat more fruits and vegetables and ‘Explore’ which contains other useful information like cooking tips, app suggestions and recipes. |
| **Goal Setting** | - Setting dietary goals. Participants set short term goals based on their self-selection of either predetermined generic goals which have been developed to target each of the food groups, or they can write their own goal. Participants will be instructed to base their goals on feedback from their personalized dietary report. - The food groups include vegetables and salad, fruit, dairy, breads and cereals, meat and alternatives, alcoholic beverages, fatty meats, sweetened drinks, packaged snacks, confectionary, baked sweet products, fried and takeaway food and spreads and sauces. - The listed generic goals have been designed as SMART (Specific-Measurable-Achievable-Realistic-Timely) goals. - They can select up to three goals to focus on at any one time. - At 3 and 6 months they will be prompted by email and/or text to revise and update their goals after they have received their personalized feedback report for intake over the preceding 3 months. |
| **Self-monitoring** | - Monitoring of dietary goals. Participants will be prompted by email and text to self-monitor their goals by going to their dashboard. - Using a 5-point scale from ‘Very poor’ to ‘Very good’, participants will be asked to reflect on how well they went with achieving their goals and how important their goal is to them (‘Very important to ‘Not important’). - Based on their responses, they will be provided with generic feedback, which will either direct them to update their goals or provide them with information that will support them on achieving their goals. |
